# Long-term exposure to fine particulate matter, ozone, and greenness and the risk of lung cancer: a retrospective cohort analysis within a national sample cohort

**DOI:** 10.3389/fpubh.2025.1661937

**Published:** 2025-09-30

**Authors:** Nam Eun Kim, Ji-Young Lee, Ga Young Lee, Chai Young Lee, Sojung Park, Yon Ju Ryu, Jin Hwa Lee

**Affiliations:** ^1^Division of Pulmonary and Critical Care Medicine, Department of Internal Medicine, College of Medicine, Ewha Womans University, Seoul, Republic of Korea; ^2^Inflammation-Cancer Microenvironment Research Center, College of Medicine, Ewha Womans University, Seoul, Republic of Korea

**Keywords:** particulate matter, ozone, greenness, lung cancer risk, air pollution

## Abstract

**Background and objective:**

Given the rising incidence of lung cancer among never smokers and growing concerns about environmental risk factors, this study investigated the association between long-term exposure to air pollution and greenness and the risk of lung cancer.

**Methods:**

We conducted a retrospective cohort analysis using data from the Korean National Sample Cohort (2002–2019), including 7,155 lung cancer patients and 28,620 propensity score-matched controls (matched by age, sex, and enrollment year). Long-term exposure to air pollution (quantified by PM_2.5_ and O₃ concentrations) and greenness (quantified by the normalized difference vegetation index, NDVI) was estimated based on residential area. Cox proportional hazards models were used to assess associations between exposure and lung cancer risk.

**Results:**

Among 35,775 participants, lung cancer patients had lower BMI, higher smoking exposure, lower household income, and higher comorbidity scores than controls. PM_2.5_ exposure showed a modest association with increased lung cancer risk in the highest tertile (aHR = 1.06; 95% CI = 1.01–1.13). O₃ exposure was consistently associated with elevated risk across all tertiles (aHR = 1.42; 95% CI = 1.34–1.50). Greenness exposure demonstrated a protective effect (aHR = 0.89; 95% CI = 0.86–0.91). Subgroup analyses indicated that PM_2.5_ effects were more pronounced among male never smokers, O₃ exposure was associated with higher risk in female never smokers and males overall, and NDVI showed protective associations across all subgroups.

**Conclusion:**

Long-term exposure to air pollution, particularly O₃, was significantly associated with an increased risk of lung cancer, independent of other confounders. In contrast, PM_2.5_ showed only a modest and inconsistent association, while high greenness exposure demonstrated a protective effect. These findings emphasize the need for region-specific environmental policies aimed at improving air quality and enhancing access to green spaces to reduce lung cancer risk.

## Introduction

Particulate matter (PM) is classified by aerodynamic diameter, which determines how deeply particles penetrate into the respiratory tract. Fine particles (PM_2.5_; ≤2.5 μm) can reach the alveoli and enter systemic circulation (1, 2). Long term exposure to PM_2.5_ adversely affects multiple organ systems (3), contributing to respiratory diseases such as chronic obstructive pulmonary disease (4), cardiovascular disease (5), neurodegenerative disorders (6). These health effects are mediated by biological mechanisms including oxidative stress, inflammation, DNA damage, and epigenetic alterations (5, 7–9).

Ozone (O₃), another major ambient air pollutant, is a highly reactive gas with well-documented adverse effects on both respiratory and cardiovascular health (10, 11). Large cohort studies have demonstrated that long-term exposure is associated with increased respiratory mortality, with a 10 ppb rise in ozone concentration conferring approximately a 4% higher risk of death from respiratory causes, independent of PM_2.5_ exposure (12). In addition, ozone exposure has been linked to new-onset asthma in children and exacerbation of asthma symptoms in affected individuals (13). The underlying biological mechanisms involve oxidative stress and the generation of reactive oxygen species, which damage DNA, impair antioxidant defenses, and induce chronic airway inflammation and epithelial injury (14, 15). Collectively, these processes may contribute to cellular proliferation, mutagenesis, and the initiation of lung carcinogenesis (16).

Multiple epidemiological studies have demonstrated a significant association between exposure to PM_2.5_ and elevated lung cancer risk, particularly in cases with a higher concentration and longer duration of exposure. In the European ESCAPE study, each 5 μg/m^3^ increase in PM_2.5_ concentrations was associated with an 18% increase in lung cancer risk (17). A large U. S. cohort study similarly reported increased rates of lung adenocarcinoma among never-smokers exposed to PM_2.5_ (18). In another long-term investigation with a median follow-up of 10 years, PM_2.5_ exposures was associated with a 12% increase in lung cancer risk (19). Meta-analyses further support these findings, consistently demonstrating that exposure to PM_2.5_ or PM₁₀ is linked to elevated lung cancer risk (20). Evidence from East Asia aligns with these observations; large-scale cohort studies have reported significant associations between PM_2.5_ exposures and both lung cancer incidence and mortality (21). A nationwide Chinese study also demonstrated significant associations between ambient PM_2.5_ concentrations and cause-specific mortality, including deaths from lung cancer (22). In Korea, an NHIS-based cohort study further reported elevated lung cancer mortality among individuals exposed to ozone alone or in combination with PM_2.5_ with odds ratios ranging from 1.15 to 1.27 (23).

Based on accumulating evidence, the International Agency for Research on Cancer (IARC), a specialized agency of the World Health Organization (WHO), classified outdoor air pollution as a Group 1 carcinogen in 2013, indicating sufficient evidence of carcinogenicity in humans (24). Notably particulate matter, a major component of outdoor air pollution, was evaluated separately and was also classified as carcinogenic to humans. Subsequent longitudinal cohort studies have strengthened this conclusion by providing temporal evidence of the health burden attributable to air pollution. For example, examined temporal trends in lung cancer mortality attributable to PM_2.5_ exposures in China over a 30-year span using age-period-cohort analysis, demonstrating increasing burdens in older cohorts (25). Another investigation provided longitudinal insights into the health benefits of greenness, showing that reduced mortality was partly mediated by decreases in PM_2.5_ and NO₂ exposures, thereby highlighting the complex and time-varying interactions among environmental factors (26).

Although O3 has been classified by the International Agency for Research on Cancer (IARC) as Group 3, indicating that its carcinogenicity in humans is not classifiable, emerging evidence suggests a potential association between O₃ exposure and lung cancer risk. Long-term exposure to ambient O₃ has been linked to lung tissue injury and chronic airway inflammation, processes that may increase the susceptibility to various pulmonary diseases, including cancer (14, 27). In addition, several studies have suggested that O₃ may contribute to carcinogenesis when combined with PM_2.5_, through mechanisms involving accelerated lung function decline and enhanced oxidative stress (28).

In this study, we aimed to analyze the effects of long-term exposure to two air pollutants (PM_2.5_ and O_3_) on the development of lung cancer using nationwide data from the Korean National Health Insurance Service (NHIS) between 2002 to 2019. We further assessed the potential protective effects of residential greenness and examined whether the associations of air pollutants with lung cancer differed according to smoking status and sex. We hypothesized that long-term exposure to PM_2.5_ and O₃ would be associated with an increased risk of lung cancer, whereas residential greenness would be associated with a reduced risk.

## Materials and methods

### Study population

This study conducted a retrospective analysis utilizing the National Health Insurance Service-National Sample Cohort (NHIS-NSC) database, a comprehensive dataset managed by the NHIS, which encompasses a representative sample of the Korean population. The NHIS in Korea maintains records of all covered inpatient and outpatient visits, procedures, and prescriptions. The NHIS established the target population using the National Health Information Database (NHID) in 2002 and created the NHIS-National Sample Cohort (NHIS-NSC) by randomly selecting a representative sample of 1,137,896 individuals, corresponding to approximately 2.2% of the eligible Korean population at that time. The NHIS-NSC is a nationwide, retrospective cohort that spans from 2002 to 2019.

The study population comprised adults aged 20 years or older who had undergone at least one national health examination and for whom data on smoking status were available. The primary endpoint of this study was the occurrence of lung cancer identified during the follow-up period. Lung cancer cases were classified based on the International Classification of Diseases, 10th Revision (ICD-10) code C34.x. To reduce the possibility of reverse causation, individuals with a lung cancer diagnosis before 2003 were excluded from analysis. Incident cases were defined as those with a first recorded diagnosis of lung cancer between 2004 and 2019. Cases were defined as individuals receiving a first-time diagnosis of lung cancer within the study period. For the comparison group, control subjects without lung cancer were randomly selected and matched to cases in a 1:4 ratio using propensity scores. Matching variables included age, sex, and year of cohort entry. Participants were tracked until the earliest of lung cancer diagnosis, death, or the end of 2019. Participants were censored at the time of death or at the end of the follow-up period if they did not develop lung cancer.

This study was approved by the Institutional Review Board (IRB) of Ewha Womans University Medical Center, Seoul, Republic of Korea (IRB number: SEUMC2021-08-003). The IRB waived the need to obtain informed consent considering the retrospective nature of the study. All procedures were conducted in accordance with the relevant guidelines and regulations outlined in the latest revision of the Declaration of Helsinki.

### Air pollution and green space exposure

The exposure variables used in this study were 5-year average concentrations of PM_2.5_ and O_3_ and the normalized difference vegetation index (NDVI). PM_2.5_ and O_3_ concentrations were estimated using a satellite-based spatiotemporal model based on aerosol optical depth data from the National Aeronautics and Space Administration (NASA). Estimates were calculated at a spatial resolution of 1 km × 1 km for each participant based on their residential address.

To determine the level of exposure to greenness, we used the NDVI, a satellite-derived metric that reflects vegetation density and plant health. NDVI data were collected from the Moderate Resolution Imaging Spectroradiometer (MODIS), as well as the Landsat 7 and 8 collections provided by the United States Geological Survey. The MODIS is the primary sensor for ground surface monitoring and is mounted on the Earth observation satellites Terra and Aqua. Its data are widely used to examine green space and other environmental factors. NDVI values were averaged for each participant based on the date of enrollment in the cohort. Long-term exposure to air pollution and green space was defined as the five-year average at participants’ residential addresses prior to cohort enrollment.

### Statistical analysis

Descriptive statistics are expressed as the mean (standard deviation) for continuous variables and number (%) for categorical variables. Differences between groups were analyzed using the *t*-test for continuous variables and chi-square test for categorical variables. Kaplan–Meier analysis and the log-rank test were used to evaluate differences in lung cancer incidence across exposure categories. Cox proportional hazards models were applied to estimate the effects of air pollution and greenness on lung cancer risk by adjusting for confounding factors such as age, sex, smoking status, household income, residential area, body mass index (BMI), and Charlson Comorbidity Index (CCI). Subgroup analyses were performed to evaluate the associations of PM_2.5_, O_3_, and green space exposure with lung cancer risk according to gender and smoking status, and the impact of O_3_ was further examined by residential area. Adjusted covariates were selected based on established or suspected confounders identified in previous literature and known risk factors for the outcomes. Observations with missing values for any variable were excluded to preserve data integrity and ensure the validity of the results.

To assess the potential nonlinear association between exposure and lung cancer risk, we modeled the relationship using restricted cubic splines within the Cox proportional hazards framework. Hazard ratios (HRs) and 95% confidence intervals (CIs) were estimated across the full range of exposure. To enhance the precision of individual exposure assessment to air pollution and green space, we conducted a sensitivity analysis. For workers, who may be exposed to environmental factors at both their workplace and residence, these exposures were explicitly considered. Using data from the NHIS, we compared lung cancer risk by classifying health insurance subscribers into workplace-based and community-based groups. Hazard ratios (HRs) with 95% confidence intervals (CIs) were calculated for lung cancer risk. SAS version 9.4 (SAS Institute, Cary, NC, United States) was used for large-scale data management, and R software version 4.0.3 (R Foundation for Statistical Computing, Vienna, Austria) was used for data analysis. The primary R packages utilized were survival (v3.2-7), survminer (v0.4.8), splines (included in base R 4.0.3), dplyr (v1.0.2), and ggplot2 (v3.3.2), which facilitated comprehensive survival analysis, data processing, and high-quality visualization. The significance level was set at *p* < 0.05.

## Results

**Figure 1:**
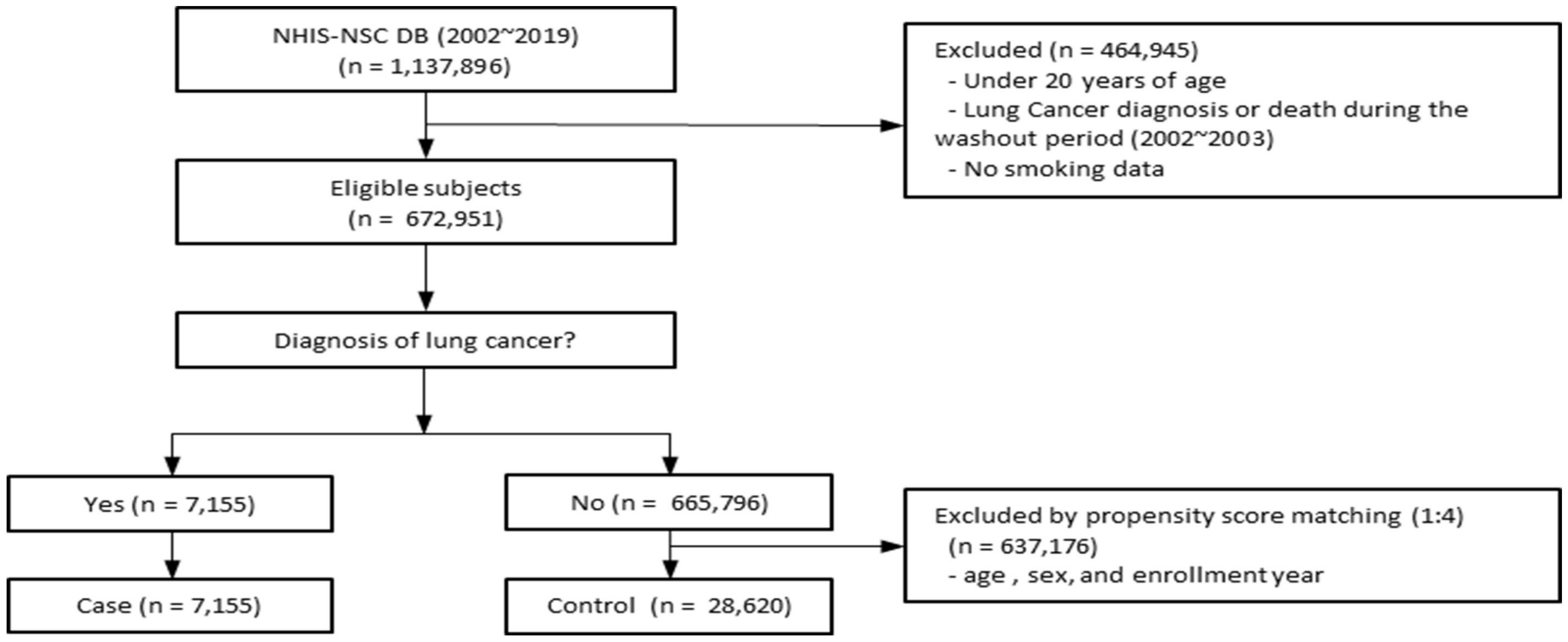
Flow diagram for selection of the study population. This figure illustrates the selection of study subjects from the NHIS-NSC database (2002–2019). After applying exclusion criteria and propensity score matching, the final cohort comprised 7,155 lung cancer cases and 28,620 matched controls. NHIS-NSC, National Health Insurance Service–National Sample Cohort.

We identified 672,951 individuals aged ≥20 years who underwent health examinations between 2002 and 2019 in the NHIS-NSC database. After excluding participants younger than 20 years, those diagnosed with lung cancer or who died during the 2002–2003 washout period, and those with missing smoking or BMI data, 7,155 patients with lung cancer remained. Using 1:4 propensity score matching, we selected 28,620 controls without lung cancer, yielding a final analytic cohort of 35,775 participants (Figure 1). The standardized mean differences for the propensity score matching variables, including age, sex, and enrollment year, were all below 0.1, indicating adequate covariate balance between the groups. A Love plot illustrating the covariate balance before and after matching is presented in the Supplementary Figure S1. Time-to-event analyses were conducted using a stratified Cox proportional hazards regression model based on the propensity score matching. The matched set ID was specified as strata, allowing the baseline hazard to vary across matched sets.

Baseline characteristics are summarized in Table 1. Compared with controls, patients with lung cancer had lower mean BMI, were more often current smokers with higher pack-years, and had lower household income, higher comorbidity scores, and were more likely to live in rural areas, and have higher CCI scores. The mean (standard deviation, SD) exposure levels for PM_2.5_, O₃, and greenness (NDVI) were 29.01 (2.42) μg/m^3^, 34.79 (3.41) ppb, and 0.16 (0.11), respectively (Supplementary Table S1). Correlation analysis of PM_2.5_, O_3_, and NDVI showed that PM_2.5_ and O_3_ showed a weak positive correlation, and PM_2.5_ and NDVI showed a weak negative correlation. There was almost no correlation between O_3_ and NDVI (Supplementary Figure S2).

**Table 1:** Baseline characteristics.

Characteristics	Without lung cancer (*n* = 28,620)	Lung cancer (*n* = 7,155)	*P*-value^†^
Age (years)*	76.7 ± 11.7	76.8 ± 11.9	0.338
20 ~ 59	2,599 (9.1)	649 (9.1)	0.995
60 ~ 70	13,637 (47.6)	3,414 (47.7)	
>71	12,384 (43.3)	3,092 (43.2)	
Men	19,300 (67.4)	4,826 (67.5)	0.982
BMI (kg/m^2^)	24.1 ± 0.2	23.4 ± 0.4	<0.001
Smoking status			
Never	15,858 (55.4)	3,562 (49.8)	<0.001
Former	9,494 (33.2)	1,935 (27.0)	
Current	3,268 (14.4)	1,658 (23.2)	
Smoking (pack-years)	8.0 ± 17.5	13.7 ± 22.6	<0.001
Household income			
<$ 2000	7,347 (26.5)	2,076 (30.0)	<0.001
$ 2000–5,000	11,955 (43.1)	2,909 (42.1)	
>$ 5,000	8,452 (30.4)	1,926 (27.9)	
Residential area			
Metropolitan	14,297 (50.0)	3,036 (42.4)	<0.001
Urban	11,089 (38.7)	2,838 (39.7)	
Rural	3,234 (11.3)	1,281 (17.9)	
CCI			
0	16,796 (91.6)	6,352 (88.8)	<0.001
≥1	1,510 (8.4)	803 (11.2)	

^†^ Two-sided chi-square and *t*-test where appropriate. * The median age was 77 years in both groups, with an interquartile range of about 69 to 85 years, under the assumption of approximate normality. Standardized mean differences for age and age categories were all <0.01.

**Figure 2:**
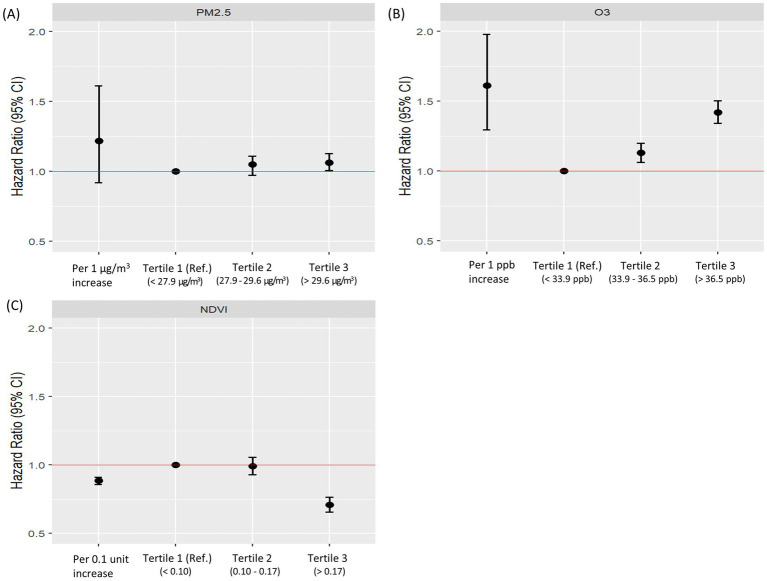
Adjusted hazard ratios for lung cancer risk according to tertiles of PM_2.5_, O₃, and NDVI. This figure shows adjusted hazard ratios (aHRs) and 95% confidence intervals (CIs) for lung cancer risk according to tertiles of **(A)** PM_2.5_, **(B)** O₃, and **(C)** NDVI. “All” represents the overall association in the total study population. “T1 (Ref.)” indicates the reference group (lowest tertile), while T2 and T3 indicate the middle and highest tertiles, respectively. The red horizontal line denotes aHR = 1.0 (the null value). Adjusted hazard ratios were adjusted for age, sex, smoking status, body mass index, household income level, residential area, and Charlson comorbidity index. aHR, adjusted hazard ratio; BMI, body mass index; CCI, Charlson Comorbidity Index; NDVI, normalized difference vegetation index.

Significant associations were identified between lung cancer risk and exposures to air pollution and greenness (Figure 2). After adjusting for major covariates, O_3_ was associated with increased lung cancer risk (aHR = 1.612; 95% CI = 1.297–1.978), and the NDVI demonstrated a protective effect (aHR = 0.885; 95% CI = 0.858–0.912). When exposures were categorized into tertiles, PM_2.5_ was significantly associated with increased lung cancer risk in the 3^rd^ tertile (aHR = 1.064; 95% CI = 1.006–1.127). O_3_ showed a positive association across all tertiles (aHR = 1.421; 95% CI = 1.342–1.503). A high NDVI indicated significant protective effects in the 3^rd^ tertile (aHR = 0.709; 95% CI = 0.658–0.764). Hazard ratios for exposure by tertiles of air pollution and NDVI are provided in the Supplementary Table S2.

**Figure 3:**
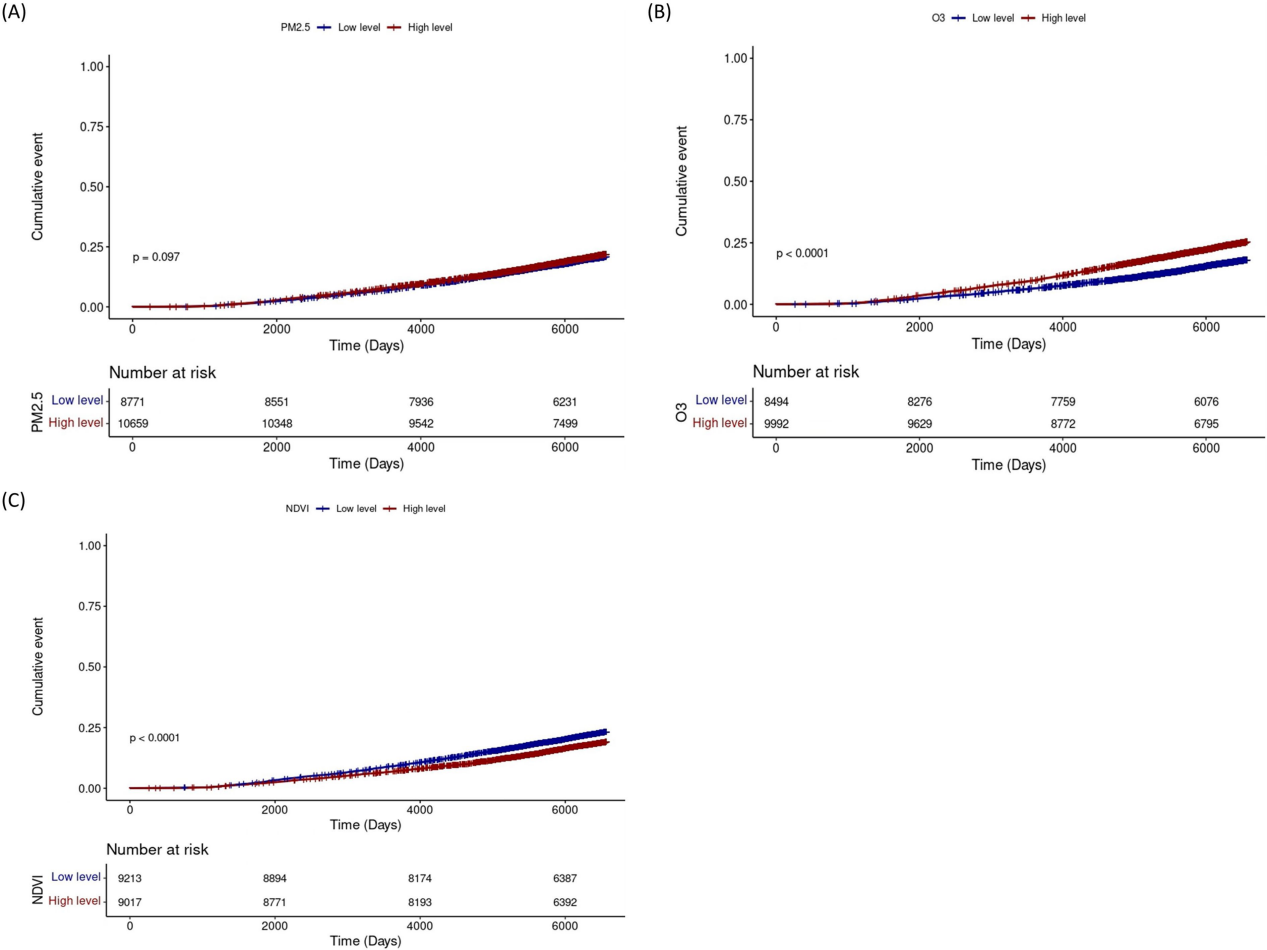
Cumulative incidence of lung cancer according to levels of PM_2.5_, O₃, and NDVI. Cumulative incidence curves for lung cancer according to low (blue) and high (red) levels of **(A)** PM_2.5_, **(B)** O₃, and **(C)** NDVI. The y-axis represents the cumulative event probability, and the x-axis shows follow-up time (days). Numbers at risk at each time point are indicated below each panel. *p*-values compare incidence between exposure groups. NDVI, Normalized Difference Vegetation Index.

Kaplan–Meier analysis demonstrated higher cumulative incidence of lung cancer with high O₃ exposure and with low NDVI (both *p < 0.001*) (Figure 3). In subgroup analyses, PM_2.5_ exposure was associated with increased lung cancer risk particularly among male never smokers and ex-smokers. O_3_ exposure showed a significant association with lung cancer risk among all male subjects and female never smokers, and the NDVI demonstrated the protective effects of greenness exposure regardless of sex and smoking status (Table 2). Results stratified by IQR increases in air pollution and NDVI, respectively, are presented in the Supplementary Table S3. There is a trend toward an increased risk of lung cancer associated with ozone exposure in rural areas, and the difference in effect between regions is of borderline statistical significance (Supplementary Figure S3).

**Table 2:** Subgroup analysis of lung cancer risk associated with air pollution and greenness exposure stratified by sex and smoking status.

	Male		Female	
	HR (95% CI)	aHR^†^ (95% CI)	HR (95% CI)	aHR^†^ (95% CI)
PM_2.5_				
Never smoker	**1.189 (1.031–1.372)**	**1.193 (1.035–1.376)**	0.924 (0.824–1.037)	0.957 (0.853–1.073)
Ex-smoker	**1.145 (1.011–1.297)**	**1.138 (1.004–1.289)**	1.283 (0.656–2.507)	1.536 (0.778–3.036)
Current smoker	1.064 (0.928–1.220)	1.071 (0.934–1.229)	0.553 (0.333–1.002)	0.617 (0.345–1.103)
O_3_				
Never smoker	**1.687 (1.442–1.973)**	**1.594 (1362–1.866)**	**1.044 (1.030–1.059)**	**1.358 (1.210–1.526)**
Ex-smoker	**1.414 (1.244–1.606)**	**1.399 (1.231–1.591)**	0.822 (0.374–1.805)	0.926(0.413–2.075)
Current smoker	**1.563 (1.354–1.803)**	**1.426 (1.235–1.646)**	**1.856 (1.101–3.130)**	**1.783 (1.054–3.016)**
NDVI				
Never smoker	**0.088 (0.077–0.102)**	**0.089 (0.077–0.103)**	**0.084 (0.074–0.095)**	**0.084 (0.074–0.095)**
Ex-smoker	**0.084 (0.074–0.096)**	**0.085 (0.075–0.098)**	**0.046 (0.017–0.122)**	**0.041 (0.014–0.123)**
Current smoker	**0.094 (0.080–0.111)**	**0.093 (0.079–0.109)**	**0.132 (0.078–0.221)**	**0.115 (0.065–0.205)**

^†^ Adjusted hazard ratios (aHRs) were adjusted for age, body mass index (BMI), household income, residential area, and * Charlson Comorbidity Index (CCI). * PM_2.5_ and O_3_ were modeled per 1 μg/m^3^ and 1 ppb increase, respectively, and NDVI per 0.1-unit increase. The bold values denote statistical significance.

The dose–response analysis demonstrated a nonlinear association between exposure and lung cancer risk. To further elucidate this relationship, we applied a restricted cubic spline model, and the resulting spline-based hazard ratio curve is presented in the Supplementary Figure S4. The curve indicates that the HR increases above a certain exposure level. The analysis of air pollution and green space exposure in relation to lung cancer risk between workplace-based and community-based groups yielded comparable results, with no statistically significant interactions observed upon inclusion of interaction terms in the model (Supplementary Table S4).

## Discussion

Overall, long-term PM_2.5_ exposure was not significantly associated with lung cancer risk; however, participants in the highest tertile of exposure showed a 6.4% higher risk compared with those in the lowest tertile. In contrast, O₃ exposure was associated with a 61.2% increased risk of lung cancer, even after adjustment for potential confounding factors. Exposure to residential greenness, measured by the NDVI, was associated with a 12% lower risk of lung cancer, with the strongest protective effects observed in the highest tertile. A clear dose–response relationship was observed for PM_2.5_, O₃, and greenness, with more pronounced effects at higher exposure levels.

Subgroup analyses indicated that never-smoking men were particularly susceptible to PM_2.5_ exposure. This may reflect biological factors, such as sex-specific differences in pulmonary responses or the lack of adaptive mechanisms that could be present in smokers, although further research is needed to clarify this vulnerability (29). Previous studies have also reported that lung cancer risk among never smokers is influenced by ambient air pollution (30). For instance, the AHSMOG-2 cohort, predominantly composed of never smokers, demonstrated an elevated risk of lung cancer associated with PM_2.5_ exposure among individuals with long-term residence or greater time spent outdoors, indicating a dose–response relationship (31). In contrast, this association was not observed among current smokers, likely because the strong carcinogenic effect of smoking may mask the association with PM_2.5_ exposure. Consistent with this, a meta-analysis reported that the effect of PM_2.5_ was attenuated after adjusting for smoking status (32).

Although recent studies have reported significant associations between PM_2.5_ exposure and lung cancer development among female never smokers, this association was not observed in our study. This discrepancy may be explained by sex-specific differences in susceptibility and exposure levels. For example, one cohort study found that males were more susceptible to lung cancer at lower PM_2.5_ concentrations (0–35 μg/m^3^), whereas females demonstrated greater susceptibility at higher concentrations (35–75, 75–115, and 115–150 μg/m^3^) (33). Similarly, a study conducted in Taiwan reported that residential PM_2.5_ exposure above 30 μg/m^3^ was associated with an increased risk of lung adenocarcinoma among females (34). In our cohort, the mean PM_2.5_ concentration was 29.01 μg/m^3^, which falls within the range associated with increased susceptibility among males but not females.

PM_2.5_ has been classified as a Group 1 carcinogen by the International Agency for Research on Cancer (IARC), a specialized agency of the World Health Organization (WHO), with proposed mechanisms involving oxidative stress, chronic inflammation, DNA damage, and epigenetic alterations. A dose–response relationship between PM_2.5_ exposure and lung cancer risk has been consistently demonstrated in epidemiological studies (35, 36). Although evidence regarding O₃ remains inconclusive, our analyses showed that O₃ was more strongly associated with lung cancer risk than PM_2.5_, warranting consideration of several possible explanations.

First, subgroup analyses revealed that O₃ exposure was significantly associated with increased lung cancer risk in all male participants regardless of smoking status, and in female never and current smokers, but not in former smokers. Mechanistically, unlike PM_2.5_, which can reach the peripheral alveoli, O₃ primarily affects the airway epithelium (16), leading to localized oxidative injury and chronic inflammation. Smokers, who often have pre-existing airway inflammation, may therefore exhibit heightened susceptibility to O₃ exposure. In addition, this increased risk may also reflect behavioral factors, as males generally spend more time outdoors, thereby increasing cumulative O₃ exposure.

Second, recent trends suggest that O₃ exposure may pose a greater health risk compared with PM_2.5_ exposure. In Korea, as well as in North America and Europe, nationwide air pollution control policies have reduced the concentrations of major pollutants, including PM_2.5_ (37). According to data from Statistics Korea, mean PM_2.5_ levels decreased from 26.1 μg/m^3^ in 2015 to 23.6 μg/m^3^ in 2019 (38). In contrast, annual O₃ concentrations increased by approximately 13 ppb, corresponding to a 42% rise over a similar period, with higher levels in rural compared with urban areas (39). A Chinese study quantifying PM_2.5_-O₃ interactions similarly reported a 25.9% reduction in the health burden attributable to PM_2.5_ but an 11.8% increase in the burden attributable to O₃, primarily affecting cardiovascular, cerebrovascular, and respiratory diseases (40). Consistent with these findings, lung cancer incidence in our study was higher in rural areas. Although rural regions generally contain more green space, ambient O₃ levels are often elevated in such areas. In urban environments, nitric oxide (NO) emitted from traffic and industrial sources reacts with O₃, leading to reduced ambient concentrations. In contrast, in rural areas, O₃ precursors such as NO₂ can be transported by wind and undergo photochemical reactions more readily, resulting in higher O₃ concentrations (39).

Lastly, global warming has been suggested to contribute to a climate penalty effect, characterized by elevated O₃ formation and adverse health outcomes. Mechanistically, higher temperatures facilitate photochemical reactions that increase ambient O₃ concentrations. Biologically, chronic O₃ exposure is associated with sustained airway inflammation and oxidative stress, which may promote carcinogenesis through DNA damage (41). From a public health perspective, vulnerable populations—including the older adult(s), socioeconomically disadvantaged individuals, and those with limited access to cooling resources—may be at heightened risk of lung cancer during periods of elevated temperature and O₃ levels (42).

In contrast to the adverse effects of air pollution, green environments may act as a protective factor against lung cancer. In our study, higher NDVI values were associated with reduced lung cancer risk, independent of sex and smoking status. Consistent with these findings, a meta-analysis reported significant reductions in lung cancer incidence and mortality with greater exposure to greenness (43). Moreover, another large-scale meta-analysis demonstrated that green space exerts a protective effect on respiratory health through multiple pathways, including improved air quality, reduced heat exposure, alleviated stress and inflammation, increased physical activity, and enhanced immune function (44).

The protective role of greenness has also been observed in relation to air pollution–related outcomes. For example, a previous study reported that green space exposure was associated with reduced PM_2.5_-related mortality, with urban residents experiencing greater benefits than rural residents (45). In our study, however, the incidence of lung cancer was higher in rural populations. This finding underscores the need for region-specific greening strategies, particularly because rural areas often experience elevated O₃ levels due to long-range transport of precursors and enhanced photochemical reactions (38). Conversely, in urban settings, reductions in NOₓ emissions can paradoxically increase O₃ concentrations by reducing the scavenging of O₃ by freshly emitted NO, as illustrated in a case study from Zaragoza, Spain (46).

This study has several limitations. First, because the study population was restricted to Korea, regional differences in air pollutant composition, climate, and urban planning may limit the generalizability of our findings. For example, the toxicity of PM_2.5_ can vary depending on dominant emission sources such as coal combustion or traffic emissions (47). O₃ levels are influenced by meteorological and climatic conditions (48), while access to green spaces differs substantially across countries (49). Therefore, multi-region cohort studies and meta-analyses are needed to confirm the applicability of our results in diverse environmental contexts.

Second, exposure estimates for PM_2.5_, O₃, and greenness were derived from satellite-based models linked to residential addresses, which may not fully capture individual exposure variability, including workplace environments and indoor air quality.

Third, other air pollutants such as NO₂ were not considered, which could confound the observed associations. Future studies incorporating multi-pollutant models are needed to better reflect real-world exposure conditions and to disentangle the independent and interactive effects of multiple pollutants on lung cancer risk. Such approaches are critical for advancing environmental epidemiology and informing effective public health policies (50, 51).

Fourth, residual confounding cannot be excluded. Unmeasured factors such as occupational exposures, lifestyle characteristics, dietary factors, and comorbidities may have influenced the results.

Fifth, NDVI reflects vegetation density but does not account for actual accessibility, quality, or individual utilization of green spaces (52). Future research should consider alternative metrics, such as proximity-based or quality-adjusted measures, to more accurately assess individuals’ true exposure to green environments.

## Conclusion

In conclusion, while PM_2.5_ has been classified as a Group 1 carcinogen by the International Agency for Research on Cancer (IARC), our findings indicate that long-term ozone exposure may represent an independent and underappreciated risk factor for lung cancer. Given anticipated changes in climate and atmospheric composition leading to rising and fluctuating ozone levels, ozone should be considered not only a short-term respiratory irritant but also a potential long-term contributor to lung carcinogenesis. Moreover, increasing access to urban green spaces may help mitigate lung cancer risk. Targeted early detection and prevention programs, particularly for high-risk groups such as never smokers and residents of high-ozone regions, may be warranted based on environmental exposure data. Finally, further research is needed to elucidate the biological pathways underlying ozone-related carcinogenesis, to examine interactions between PM_2.5_ and O₃, and to clarify the protective role of greenness across diverse populations and urban settings. Collectively, these findings provide evidence to inform global environmental interventions aimed at reducing the burden of lung cancer attributable to air pollution.

## Data Availability

The original contributions presented in the study are included in the article/Supplementary material, further inquiries can be directed to the corresponding author.
