# Supplementary material for: Long-term exposure to fine particulate matter, ozone, and greenness and the risk of lung cancer: a retrospective cohort analysis within a national sample cohort
Source: Front Public Health. 2025 Sep 30;13:1661937. doi: 10.3389/fpubh.2025.1661937 (PMC12518234; doi:10.3389/fpubh.2025.1661937)
Supplement: Supplementary file 1 [file Data_Sheet_1.docx]

**Supplementary Materials**

**Long-term exposure to fine particulate matter, ozone, and greenness and the risk of lung cancer: a population-based case-control study**

**Table of Contents**

**Table S1. Distributions of prenatal PM_2.5_/O_3_ exposure, and greenness levels in residential areas**

**Table S2. The risk of lung cancer with air pollution and green space exposures of all subjects**

**Table S3. Subgroup analysis of lung cancer risk associated with air pollution and greenness exposure per interquartile range increase, stratified by sex and smoking status**

**Table S4. Risk of lung cancer associated with air pollution and green space exposure in workplace- and community-based groups**

**Figure S1. Love plot showing standardized mean differences for covariates before and after propensity score matching.**

**Figure S2. Pearson correlation heatmap between PM_2.5_, O_3_, and NDVI.**

**Figure S3. Urbanicity-stratified hazard ratios for lung cancer survival associated with ozone exposure.**

**Figure S4. Nonlinear associations between environmental exposures and lung cancer risk.**

**Table S1.** Distributions of prenatal PM_2.5_/O_3_ exposure, and greenness levels in residential areas

| Exposure | Mean (SD) | Minimum | Percentiles | | | Maximum |
| --- | --- | --- | --- | --- | --- | --- |
|  |  |  | 25 | 50 | 75 |  |
| PM_2.5_ (μg/m^3^) | 29.01 (2.42) | 21.41 | 27.46 | 28.72 | 30.35 | 42.07 |
| O_3_ (ppb) | 34.79 (3.41) | 27.88 | 32.45 | 35.08 | 37.92 | 41.56 |
| NDVI | 0.16 (0.11) | -0.06 | 0.07 | 0.12 | 0.20 | 0.65 |

Abbreviations: SD, standard deviation.

**Table S2**. The risk of lung cancer with air pollution and green space exposures of all subjects

|  | HR (95% CI) | aHR* (95% CI) |
| --- | --- | --- |
| PM_2.5_ | 1.146 (0.869 – 1.512) | **1.153 (0.872 – 1.525)** |
| Tertile 1 | Reference | Reference |
| Tertile 2 | 1.047 (0.989 – 1.108) | 1.037 (0.979 – 1.098) |
| Tertile 3 | 1.058 (1.000 – 1.120) | 1.063 (1.003 - 1.127) |
| O_3_ | 1.717 (1.310 – 2.250) | 1.780 (1.358 – 2.335) |
| Tertile 1 | Reference | Reference |
| Tertile 2 | 1.144 (1.077 – 1.215) | 1.158 (1.090 – 1.230) |
| Tertile 3 | 1.497 (1.414 – 1.585) | 1.495 (1.412 – 1.582) |
| NDVI | 0.964 (0.872 – 0.989) | 0.970 (0.894 – 0.991) |
| Tertile 1 | Reference | Reference |
| Tertile 2 | 0.899 (0.597 – 1.052) | 0.874 (0.648 – 1.101) |
| Tertile 3 | 0.801 (0.769 – 0.943) | 0.778 (0.699 – 0.870) |

* Adjusted hazard ratios were adjusted for age, sex, smoking status, body mass index, household income level, residential area, and Charlson comorbidity index. Abbreviations: HR, hazard ratio; NDVI, normalized difference vegetation index.

**Table S3.** Subgroup analysis of lung cancer risk associated with air pollution and greenness exposure per interquartile range increase, stratified by sex and smoking status.

|  | Male | | Female | | |
| --- | --- | --- | --- | --- | --- |
|  | HR (95% CI) | aHR^†^ (95% CI) | | HR (95% CI) | aHR^†^ (95% CI) |
| PM_2.5_ |  |  | |  |  |
| Never smoker | **1.822 (1.093 - 2.596)** | **1.859 (1.108 - 2.642)** | | 0.783 (0.594 - 1.113) | 0.887 (0.666 - 1.231) |
| Ex-smoker | **1.430 (1.033 - 2.169)** | **1.393 (1.012 - 2.120)** | | 2.044 (0.328 - 4.920) | 2.919 (0.488 - 5.111) |
| Current smoker | 1.168 (0.752 - 1.998) | 1.188 (0.769 - 2.027) | | 0.217 (0.065 - 1.006) | 0.276 (0.074 - 1.284) |
| O_3_ |  |  | |  |  |
| Never smoker | **17.471 (7.405-34.148)** | **12.812 (5.419-30.331)** | | **1.266 (1.175 - 1.368)** | **5.333 (2.837 - 1.526)** |
| Ex-smoker | **6.309 (6.042 - 6.588)** | **6.158 (5.916 - 6.410)** | | 0.361 (0.006 -21.562) | 0.670 (0.010 -38.514) |
| Current smoker | **10.200 (4.835-21.438)** | **6.330 (2.997-13.349)** | | **22.024(1.618-99.415)** | **18.020(1.301-98.549)** |
| NDVI |  |  | |  |  |
| Never smoker | **0.729 (0.717 - 0.743)** | **0.730 (0.717 - 0.744)** | | **0.725 (0.713 -0.736)** | **0.725 (0.074 - 0.095)** |
| Ex-smoker | **0.725 (0.713 - 0.739)** | **0.726 (0.714 - 0.739)** | | **0.670 (0.589 - 0.761)** | **0.660 (0.589 - 0.761)** |
| Current smoker | **0.735 (0.720 – 0.751)** | **0.734 (0.719 - 0.750)** | | **0.769 (0.718 - 0.822)** | **0.755 (0.701 - 0.814)** |

† Adjusted hazard ratios (aHRs) were adjusted for age, body mass index (BMI), household income, residential area, and ***** Charlson Comorbidity Index (CCI).

**Table S4.** Risk of lung cancer associated with air pollution and green space exposure in workplace- and community-based groups

|  | Lung cancer, adjusted HR* (95% CI) | | |
| --- | --- | --- | --- |
|  | PM_2.5_ (μg/ m^3^) | O_3_ (ppb) | NDVI |
| Workplace- based group | 1.149 (0.812 – 1.785) | 1.791 (1.301 – 2.314) | 0.952 (0.901 – 0.993) |
| Community-based group | 1.153 (0.880 – 1.628) | 1.762 (1.421 – 2.511) | 0.980 (0.894 - 0.990) |
| Interation term (Exposure x Group) p-value | 0.624 | 0.151 | 0.257 |

* Adjusted hazard ratios were adjusted for age, sex, smoking status, body mass index, household income level, residential area, and Charlson comorbidity index. Abbreviations: HR, hazard ratio; NDVI, normalized difference vegetation index.


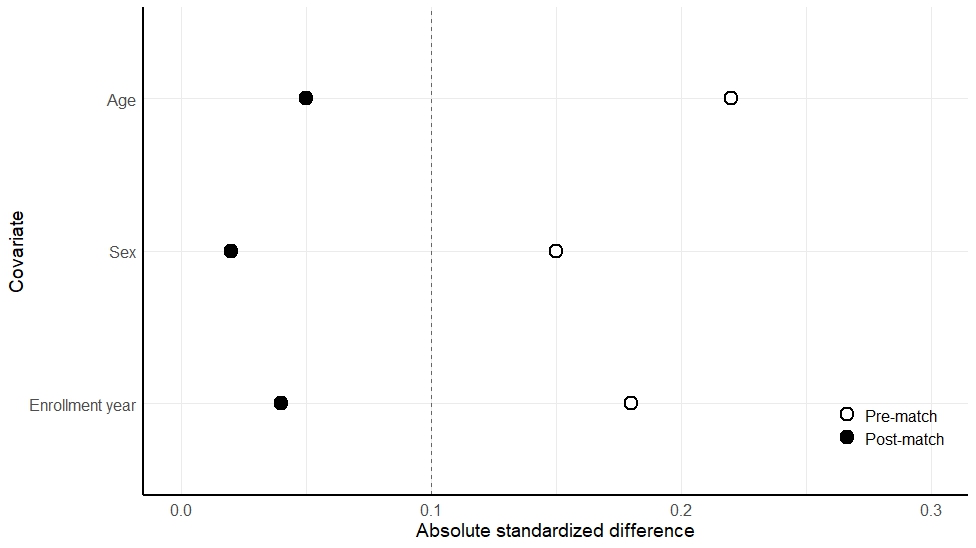


**Figure S1.** Love plot showing standardized mean differences for covariates before and after propensity score matching. Values less than 0.1 indicate adequate covariate balance. Key variables include age, sex, enrollment year.


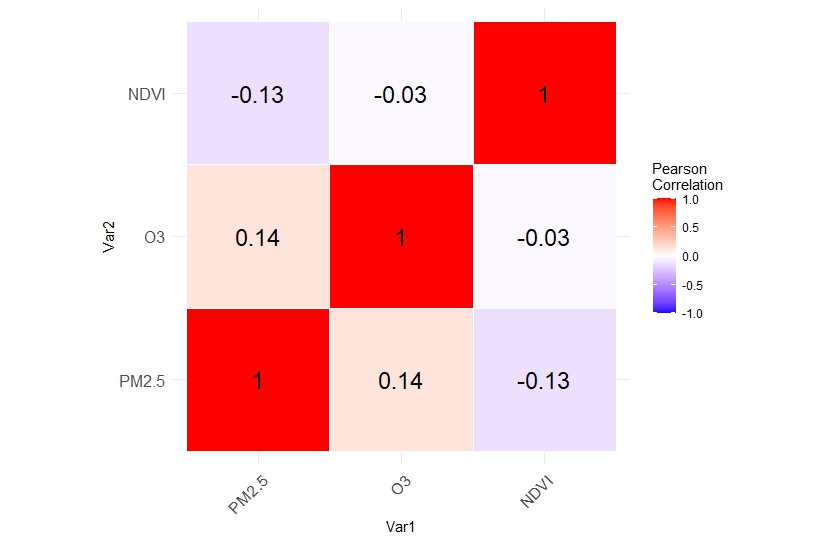


**Figure S2.** Pearson Correlation Heatmap between PM_2.5_, O_3_, and NDVI. This heatmap visualizes the Pearson correlation coefficients among PM_2.5_, O_3_, and NDVI. Color intensity represents the strength and direction of the correlation, with annotated values indicating the coefficient for each variable pair. PM_2.5_ and O_3_, showed a weak positive correlation (r = 0.14), while PM_2.5_, and NDVI exhibited a weak negative correlation (r = -0.13). The correlation between O₃ and NDVI was negligible (r = -0.03).


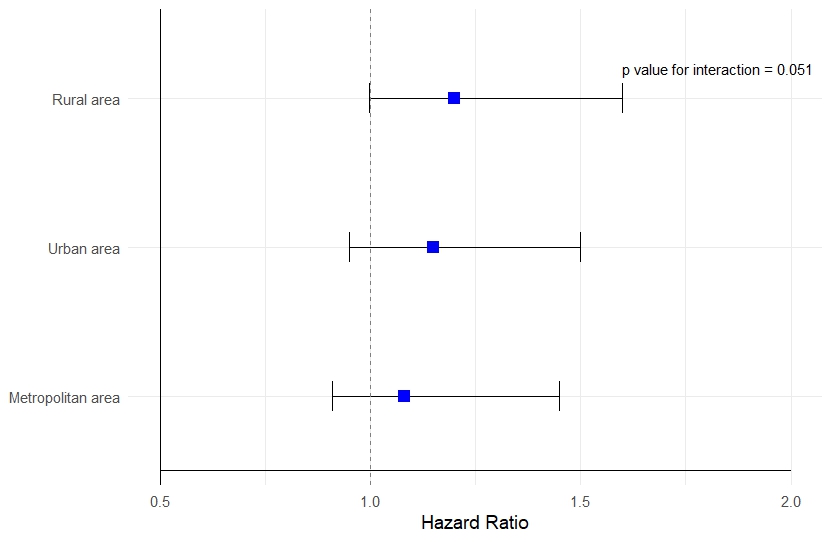


**Figure S3.** Urbanicity-Stratified Hazard Ratios for Lung Cancer Survival Associated with Ozone Exposure. This forest plot displays hazard ratios (HRs) and 95% confidence intervals (CIs) for lung cancer survival associated with ozone exposure, stratified by urbanicity categories: Metropolitan area, Urban area, and Rural area. The dashed vertical line indicates a hazard ratio of 1 (no effect). The interaction p-value (0.051) suggests a borderline statistically significant difference in ozone effects across urbanicity strata.


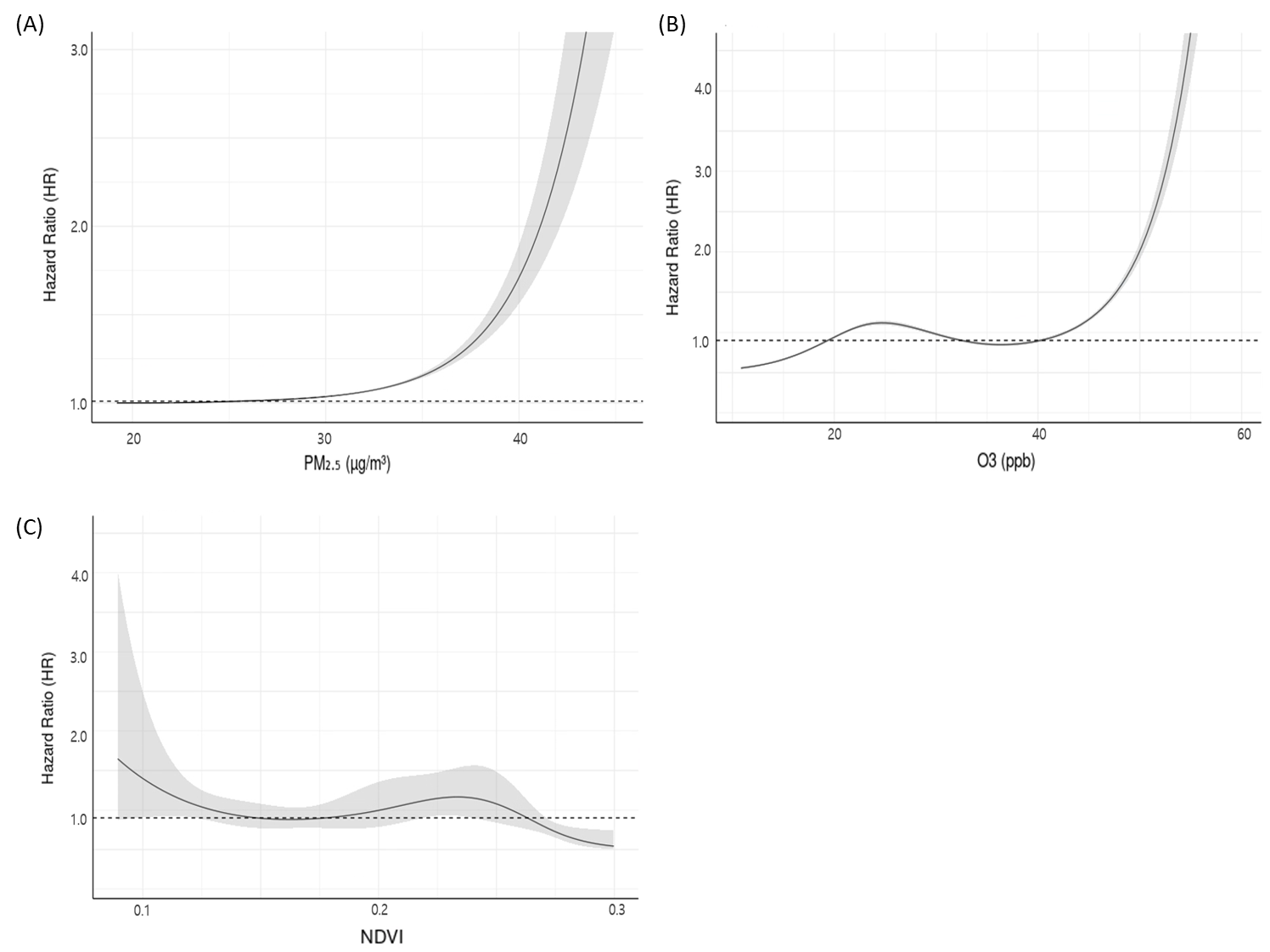


Figure S4. Nonlinear associations between environmental exposures and lung cancer risk. The figure shows exposure–response relationships between (A) PM2.5 (μg/m³), (B) O3 (ppb), and (C) NDVI and the risk of lung cancer, modeled using natural cubic splines. Solid lines represent adjusted hazard ratios (HRs), and shaded areas denote 95% confidence intervals. The models were adjusted for age, sex, smoking status, body mass index, household income level, residential area, and Charlson comorbidity index. Abbreviation: NDVI, normalized difference vegetation index.
